# Adding epitope compatibility to deceased donor kidney allocation criteria: recommendations from a pan-Canadian online public deliberation

**DOI:** 10.1186/s12882-023-03224-z

**Published:** 2023-06-09

**Authors:** Louisa Edwards, Colene Bentley, Michael Burgess, Ruth Sapir-Pichhadze, David Hartell, Paul Keown, Stirling Bryan

**Affiliations:** 1 School of Population & Public Health, University of British Columbia (UBC), 717 - 828 West 10th Avenue, Research Pavilion, Vancouver, BC V5Z 1M9 Canada; 2 Centre for Clinical Epidemiology and Evaluation, Vancouver Coastal Health Research Institute, Vancouver, Canada; 3 BC Cancer, Vancouver, Canada; 4 W. Maurice Young Centre for Applied Ethics, UBC, Vancouver, Canada; 5 Division of Nephrology, Department of Medicine, McGill University, Montreal, Canada; 6 Centre for Outcomes Research & Evaluation, Research Institute of the McGill University Health Centre, Vancouver, Canada; 7 Hartell Consulting, Ottawa, Canada; 8 Department of Medicine, UBC, Vancouver, Canada; 9 Immune Centre of BC, Vancouver Coastal Health, Vancouver, Canada

## Abstract

**Background:**

The widening supply–demand imbalance for kidneys necessitates finding ways to reduce rejection and improve transplant outcomes. Human leukocyte antigen (HLA) epitope compatibility between donor and recipient may minimize premature graft loss and prolong survival, but incorporating this strategy to deceased donor allocation criteria prioritizes transplant outcomes over wait times. An online public deliberation was held to identify acceptable trade-offs when implementing epitope compatibility to guide Canadian policymakers and health professionals in deciding how best to allocate kidneys fairly.

**Methods:**

Invitations were mailed to 35,000 randomly-selected Canadian households, with over-sampling of rural/remote locations. Participants were selected for socio-demographic diversity and geographic representation. Five two-hour online sessions were held from November–December 2021. Participants received an information booklet and heard from expert speakers prior to deliberating on how to fairly implement epitope compatibility for transplant candidates and governance issues. Participants collectively generated and voted on recommendations. In the final session, kidney donation and allocation policymakers engaged with participants. Sessions were recorded and transcribed.

**Results:**

Thirty-two individuals participated and generated nine recommendations. There was consensus on adding epitope compatibility to the existing deceased donor kidney allocation criteria. However, participants recommended including safeguards/flexibility around this (e.g., mitigating declining health). They called for a transition period to epitope compatibility, including an ongoing comprehensive public education program. Participants unanimously recommended regular monitoring and public sharing of epitope-based transplant outcomes.

**Conclusions:**

Participants supported adding epitope compatibility to kidney allocation criteria, but advised safeguards and flexibility around implementation. These recommendations provide guidance to policymakers about incorporating epitope-based deceased donor allocation criteria.

**Supplementary Information:**

The online version contains supplementary material available at 10.1186/s12882-023-03224-z.

## Background

Kidney disease afflicts one in 10 Canadians [1], has increased by 31% since 2011 [1], and was the 10^th^leading cause of death in Canada in 2019 [2]. Transplantation is the preferred treatment for kidney failure; it improves survival and quality of life for the patient [3], and decreases annual treatment costs by 50–70% compared with facility-based dialysis [4]. Without a suitable living donor (friend, family member, anonymous donor, or swapping an incompatible living donor with another incompatible donor/recipient pair through a Kidney Paired Donation program [5, 6]), the patient would need to wait for a deceased donor kidney to become available. However, the number of patients who need a transplant exceed the kidneys available, and the median wait time for a deceased donor kidney in Canada now approaches four years [1]. Initiatives to increase donation exist, such as presumed consent, whereby people need to opt-out rather than opt-in to donate organs, and this has resulted in some of the highest donation rates in Europe [7] and, recently, in Nova Scotia, Canada [8]. Nevertheless, many patients require multiple transplants over their lifetime due to premature graft failure, which compounds the scarcity issue. In Canada, over 40% of deceased donor kidneys are lost by 10 years [9] and more than half by 15 years [10] post-transplant. The rate of graft failure is even higher in the US, where ~ 50% are lost by 10 years post-transplant [11]. While many factors jeopardize the transplanted organ, the leading cause of graft loss is antibody-mediated or mixed rejection [12]. Therefore, effective strategies to reduce this kind of rejection and improve post-transplant outcomes are urgently needed.

One possibility is to consider donor-recipient matching to achieve epitope (or molecular) compatibility, which could be incorporated into deceased donor kidney allocation. Epitope compatibility is assessed by comparing targeted segments of human leukocyte antigens (HLA) in donors and potential recipients; the degree of compatibility is determined by the number and identity of molecular mismatches, as well as their demonstrated capacity to induce an antibody response [13]. Studies have shown that greater epitope compatibility can reduce the risk of rejection and graft failure [14–16]. In practice, the Eurotransplant Acceptable Mismatch program, which has operated for over 25 years, has demonstrated these clinical benefits in highly sensitized patients [17, 18], while the Royal Children’s Hospital Melbourne, Australia initiated the first paediatric epitope-based system in 2014 with promising short-term results [19]. Epitope-based allocation may be particularly advantageous for younger patients, who often need more than one transplant over the course of their life. Reduction in transplant failure would maximize the availability of kidneys for other candidates, and improve the overall economic benefit transplantation [4].

Despite these potential benefits, allocation guided by epitope compatibility may affect the order in which candidates are offered kidneys and, hence, individual waiting time. This would represent an important change from the current allocation strategy in Canada, which prioritizes time on the waiting list, combined with blood group matching, verification that pre-formed donor-specific antibodies are absent, and other clinical factors (e.g., attempt to age match donors and recipients; see Additional file 1, Sects. 7–8). Including epitope compatibility would, instead, place greater priority on transplantation outcomes than on waiting time. In this way, a donated kidney would be offered to the most epitope-compatible candidate (i.e., the candidate with the best chance of improved long-term outcomes). However, it could mean that some candidates who have already spent several years on the waiting list might have to wait even longer for a compatible match. Additionally, some candidates may be missing epitopes that are common among donors and could wait an exceptionally long time for a well-matched kidney.

When considering changes to allocation criteria, medical professionals, policy experts, patients, and the public are all key stakeholders to consult. This is particularly true in single-payer healthcare systems like Canada, in which most direct healthcare costs are covered through public funding. Regarding the public, some have argued that donor organs are a community-held resource [20], in which the system is jointly reliant on the public’s willingness to donate and directly impacted by the needs of those requiring transplantation [21]. Organ donation relies on public trust in the rules and criteria governing the allocation system, and so including the public in decision-making helps to maintain trust and transparency. This may be especially poignant in the case of epitope compatibility, in which there are potential benefits and risks to changing the allocation system. Finally, public input on changing allocation criteria is also significant because kidney failure is common and, unfortunately, will ultimately impact many members of the public. Yet, a recent systematic review noted the infrequent focus on public preferences or priorities in organ allocation in research [21].

We invited members of the Canadian public to identify what is important to them about epitope compatibility, and to provide recommendations to policymakers in a public deliberation. Public deliberations provide an opportunity for citizens to be actively involved in collective decision-making [22]. In light of the COVID-19 pandemic, we adapted well-established public deliberation methods [23] to a pan-Canadian online event with five two-hour sessions spread over five weeks. Public input was sought on how best to allocate kidneys in a way that is fair to all candidates, and identifying important considerations in allocation decision-making. This paper provides details of the online public deliberation process and participant-generated recommendations, including the reasons provided in favor of, against, or when abstaining from the recommendations.

## Methods

### Public deliberations

A public deliberation is a structured discussion with 25–30 members of the public about issues that may have an important impact on society [24]. They differ from focus groups or other research methods by the extent of information provision, the depth and length of discussions over repeated interactions, and the actionable participant-generated recommendations, which are shared with policymakers [25–27]. They are often used to help resolve social dilemmas when evidence is uncertain and value judgements may conflict [24, 27]. Public deliberations are a means of developing informed, civic-minded advice from diverse members of the public. They can enable better decision-making for policymakers because they highlight the potential social and ethical implications of a policy decision amongst the public [22]. These policy decisions are based on values or making trade-offs. The goal of a public deliberation is for participants to come together as a group, learn about a specific topic, and make recommendations on what they collectively consider the best course of action to take on a particular issue. The participant-generated recommendations are voted on, and reasons underpinning the votes are documented. Voting not only provides quantification of the group’s support for the recommendations, it also enables the opportunity to discuss and revise recommendations that are not well supported. Achieving consensus in the recommendations is desirable, but when this is not possible, points of persistent disagreement are noted.

### Recruitment

Public deliberations aim to select a 'mini public' that is able to draw upon and represent different needs, views, and values [28]. This is usually done by selecting a small group of participants based on a range of life experiences, reflected in socio-demographic differences. To facilitate an open discussion and to avoid introducing hierarchy of opinions, public deliberations typically exclude those with political or direct experience with the condition of interest. Instead, those perspectives are included in developing the deliberative questions, preparatory material (e.g., information booklet), and expert speaker presentations.

We sought to recruit members of the Canadian general public for the online public deliberation. Adults (18 + years) living in Canada, fluent in English, with access to technology capable of connecting to Zoom (audio and video required), and who were able to attend all five sessions were eligible. We excluded anyone with kidney disease or those with someone close to them with kidney disease, those who worked or volunteered for a kidney disease organization, health professionals, policymakers, and lobbyists.

The main method of recruitment was by postal invitation to 35,000 random households in all provinces and territories in Canada. The number sent per province/territory was based on population Census distributions, but with 10% over-sampling in rural/remote areas. A professional direct mail company, partnered with Canada Post, conducted the mailing in October 2021. The two-page invitation package included an invitation letter and flyer, which specified the topic, dates, reimbursement ($50 CAD/session), and details about how to register interest. Additional recruitment methods included snowballing (sharing the invitation letter) and Twitter.

Those interested in participating completed an online recruitment survey, including eligibility screening questions, socio-demographic questions for selection purposes, and providing contact details for study notification. Expressions of interest were collected for ~ 7–14 days, depending on postal delivery times.

We aimed to consent 35 participants, with the hope of retaining ~ 28 participants across all five sessions. Participant selection was guided by ensuring representation from five Canadian regions (Western, Prairies, Central, Maritimes, Northern Territories) and according to key socio-demographic variables of interest (gender, age group, education, ethnicity, religion, and urban/rural location). Selected potential participants were notified by email and asked to complete an online consent form within a few days if they still wished to participate. One reminder email was sent if a selected participant did not consent within this timeframe. After obtaining consent, participants were both emailed and mailed a welcome letter, event agenda, Zoom user guide, and the information booklet to help them prepare for the deliberation.

### Providing background information

Participants in a public deliberation are supported with background information in various formats, which is intended to make them an informed public, and enables them to engage in meaningful deliberation [26]. A key source of background information is the information booklet, which provided an overview of end-stage kidney disease, treatments for it, Canada’s current kidney allocation system, and an introduction to epitope compatibility and how it could be used in kidney transplantation decision-making (see Additional file 1). The booklet was developed by the research team, underwent plain language checking, and was reviewed iteratively by experts, policymakers, and patient partners.

Another important source of information is expert speakers. In the first session, participants heard from four speakers, who were chosen to provide a range of perspectives on this topic, including a transplant nephrologist, a bioethicist, an Indigenous knowledge keeper and elder, and a patient with end-stage kidney disease (more information provided in Additional file 2). Each presentation raised some of the possible trade-offs – the potential advantages and concerns – of introducing epitope compatibility from a different perspective, with the goal of providing balanced views. It was important to have an Indigenous community leader provide a cultural lens on this discussion, given kidney disease disproportionately affects members of Indigenous communities [29], and healthcare access and perspectives of the healthcare system vary from community-to-community [30]. A participant-driven question and answer session followed the expert speaker presentations, in which one additional patient with end-stage kidney disease participated.

### Adaptations for online event

Public deliberations are usually held in person, but due to the COVID-19 pandemic, we pivoted methods to hold this event virtually over Zoom. First, instead of holding a four-day event, we reduced this to five two-hour sessions held over several consecutive weeks from November–December 2021 (see Table 1). We reasoned that longer or a greater number of sessions could be a barrier to recruitment, suffer higher rates of attrition and/or disengagement. Second, participants focused on two deliberative questions, rather than 3–5. Third, in-person deliberations usually consist of several small group discussions, but the current online deliberation included a single small group session. Fourth, policymakers, researchers, and others interested in watching public deliberations as non-interacting ‘observers’ is common and distinctive of this methodology. We opted to live-stream the event for up to 20 observers (5–10 is typical for in-person events), which meant that they viewed the session in real-time, but they were not seen by or able to interact with participants. Instead of introducing themselves, their presence was acknowledged at the start of each session and a detailed information sheet of all observers was circulated before every session.

**Table 1:** Online Public Deliberation Format

Session	Who	Focus	Research team roles
1	All participants	Expert speaker presentations: 1) transplant nephrologist, 2) bioethicist, 3) Indigenous knowledge keeper and elder, 4) transplant recipient (patient) Provide balanced information; opportunity to ask questions	Facilitator (CB), Methodological support (MB), Welcome (SB), Vote counter & participant queries (LE), Experts available (RSP, PK); IT support, second vote counter, note taker (2 additional staff)
2	Small groups (6–8 participants)	Get to know other participants Consider a broad range of perspectives (‘hopes and concerns’) Review the goals of the deliberation	Facilitator (CB), Note taker (LE); IT support (1 additional staff)
3	All participants	Discuss deliberation questions (× 2): ○ Explore different beliefs and the reasons for them ○ Construct & vote on recommendations	Facilitator (CB), Methodological support (MB), Welcome (SB), Vote counter & participant queries (LE), Expert available (RSP); IT support, second vote counter, note taker (2 additional staff)
4			
5	Policy Panelists (× 4) + All participants	Review & discuss recommendations Ask participants for clarification	Facilitator (CB), Methodological support (MB), Welcome (SB), Vote counter & participant queries (LE), Expert available (RSP); IT support, second vote counter, note taker (2 additional staff)

### Structure of the event

All sessions were led by a trained facilitator (CB) with more than a decade of experience in public deliberation. In Session 1, the four invited experts gave 8-min presentations on a range of issues about kidney transplantation (see Additional file 2), followed by questions and answers. Session 2 was the only small-group (6–8 participants) session; its goal was to identify the different perspectives, beliefs, and values of participants in a less crowded setting. Prior to the session, participants were asked to come up with at least three hopes and three concerns about adding epitope compatibility to kidney allocation criteria, which were then shared and discussed within the group. Sessions 3 and 4 were held with the full group of participants, who discussed two questions (see below) about the trade-offs of introducing epitope compatibility. Participants worked together to construct recommendations to inform epitope-based kidney allocation policies. The final session was a policy panel discussion with kidney donation and allocation policymakers from three provincial organ donation organizations (BC Transplant, Trillium Gift of Life, Transplant Quebec) and one national organization (Canadian Blood Services). Policymakers reviewed the recommendations in advance, and then shared their thoughts and asked participants for more information during Session 5. Subsequently, participants were emailed a link to an online satisfaction survey. In addition to questions about feeling engaged, heard and supported throughout the deliberation, the survey asked participants about their satisfaction with the online event format.

The research team met several times over many months with clinicians, researchers, patient partners, methodologists, bioethicists, and policymakers (BC Transplant, Trillium Gift of Life, Transplant Quebec, Canadian Blood Services) to develop and refine the two deliberation questions and a narrative scenario (see Additional file 3). These were emailed to participants (by LE) two days in advance of Sessions 3 and 4, respectively:

1. *How can epitope-based allocation be implemented in a way that is fair for transplant candidates?*
2. *What are important considerations in the way kidney allocation policies and decisions are made?*

The narrative scenario accompanied the first deliberative question to provide context around the trade-offs and implications of changing kidney allocation criteria. It described two candidates – one easy-to-match and one more difficult to match based on blood type – and described anticipated wait times and graft longevity under the current and epitope compatibility-based systems. The scenario contrasted greater certainty about the waiting time in the current system versus more certainty about receiving a kidney that functions better and for longer with epitope compatibility.

### Analysis of deliberative output

All sessions were audio- and video-recorded and transcribed verbatim. Note takers shared their computer screens via Zoom to display the hopes and concerns generated during Session 2, as well as the recommendations, votes and reasons for voting in Sessions 3–5. The focus of the current analysis is on the deliberative output [23], which consists of the recommendations, votes, and collective reasons for the votes. While the votes provide a quantitative measure of the degree of support for recommendations, the reasons behind the numbers are more significant [31]; often participants are closer (or sometimes further apart) in agreement than the numbers indicate. For instance, participants might disagree with a specific word, but otherwise agree with the intention of the recommendation. Therefore, this analysis is focused on participants’ reasons for voting, and less emphasis is given to interpreting numerical differences in votes. An in-depth qualitative analysis of the core values underpinning these recommendations was conducted and reported separately [32].

## Results

Of the 239 expressions of interest received, 91 people were potentially eligible, and 47 were invited to participate. Thirty-seven participants provided consent, but five subsequently declined, were ineligible, or non-contactable. The remaining 32 participants came from all five Canadian regions, including 13% (4/32) from rural/remote locations (see Table 2). Retention of participants across all sessions was 91% (29/32): 31 took part in Sessions 2–3, 30 in Session 4, and 29 in Session 5.

**Table 2:** Self-reported socio-demographic characteristics of deliberation participants (*N* = 32)

	N (%)
Gender	
Female	18 (56)
Male	14 (44)
Ethnic background^a^	
Arab	3 (9)
East Asian	2 (6)
Indigenous	2 (6)
Latin, South or Central American	1 (3)
South Asian	1 (3)
White	23 (72)
Region	
West Coast	5 (16)
Prairie Provinces	10 (31)
Northern Territories	1 (3)
Central Canada	11 (34)
Atlantic Provinces	5 (16)
Country of birth	
Canada	30 (94)
Other	2 (6)
Age group (years)	
18–24	4 (13)
25–34	3 (9)
35–49	9 (28)
50–64	10 (31)
65 +	6 (19)
Highest level of education attained	
High school diploma/certificate	3 (9)
College/apprenticeship (non-university)	4 (13)
Some university	8 (25)
University degree or diploma (BA/BSc level)	11 (34)
Professional or graduate degree	6 (19)
Main activity	
Working at a paid job/business	18 (56)
Retired	8 (25)
Looking for paid work	2 (6)
Going to school	2 (6)
Household work	1 (3)
Long-term illness	1 (3)
Chronic condition (personal or dependent; excluding kidney disease)	
Yes	8 (25)
No	24 (75)
Income ($ CAD)	
Less than $20,000	3 (9)
$35,000-$49,999	2 (6)
$50,000-$79,999	6 (19)
$80,000-$99,999	9 (28)
$100,000 +	12 (38)
Religion	
Christian (United, Baptist, Anglican, Catholic)^b^	17 (53)
No religion	12 (38)
Hindu	1 (3)
Aboriginal spirituality	1 (3)
Muslim	1 (3)

*Y* yes (votes in favour), *N* no (votes against), *A* abstain from voting

NB: Percentages may not always sum to 100% due to rounding

^a^Categories are based on the Canadian Census categories for ethnic origin

^b^Denominations of Christianity were asked separately, but have been grouped here for ease of presentation. Religious categories were based on the Canadian Census categories

Participants co-constructed and then voted (yes, no, or abstain) on nine recommendations (see Table 3). These were subsequently grouped into four categories: 1) Support for adding epitope compatibility; 2) Safeguards and/or flexibility needed; 3) Transition plan and period; and 4) Ongoing monitoring and assessment. Support was largely obtained for all but Recommendations 4 and 5.

**Table 3:** Recommendations and distribution of votes

Recommendations	Y	N	A
*Support for adding epitope compatibility*			
1. Epitope compatibility should be added as an additional criterion (added to the matrix) for transplant candidate selection	30	0	1
*Safeguards and/or flexibility needed*			
2. Safeguards/flexibility need to be part of epitope compatibility to promote fairness.	28	0	3
3. When epitope compatibility is being considered, we should also allow people with seriously declining health to receive less- or non-epitope matched kidneys.^a^	23	3	1
4. Quality of life should be considered as a priority.	11	12	7
5. Deteriorating health should be considered as a priority.	20	5	5
6. Epitope matching should be given high, but not absolute priority in the allocation of kidneys.	29	0	1
*Transition plan and period*			
7. There needs to be an ongoing comprehensive education program for the public, beginning with the transition to epitope matching.	27	1	2
8. There needs to be a transition period and plan before starting the epitope matching system.^b^	25	0	2
*Ongoing monitoring and assessment*			
9. Assessing epitope compatibility outcomes at least every 5 years and communicate results widely to patients, healthcare professionals, and public, whether successful or not.	29	0	0

^a^This session went overtime and 4 participants were unable to stay longer and vote

^b^This session went overtime and 3 participants were unable to stay longer and vote

### 1. Support for adding epitope compatibility

There was nearly unanimous support for adding epitope compatibility to the current deceased donor kidney allocation system (Recommendation 1). Participants understood that allocation is complex and already guided by many criteria (i.e., a “matrix”). There was a sense that fairness existed in the current system, which participants wanted to preserve. Therefore, they did not support an overhaul of the current system, but viewed epitope compatibility as another “tool in the toolkit”. Some indicated support for adding epitope compatibility because they felt this could be done flexibly. Others felt it would not make sense to ignore/disregard scientific evidence around epitope compatibility. However, there was a lot of discussion about the fact that the scientific evidence was still emerging, especially about how effective this technology might be in improving transplantation outcomes. This meant that many were not willing to more strongly advocate for changing the current system in favor of epitope-based allocation, until or unless the data showed clear, positive results. The one abstention felt that time on the waiting list should be replaced with a consideration of candidates’ health state.

### 2. Safeguards and/or flexibility needed

Participants endorsed a general need for safeguards and/or flexibility when adding epitope compatibility, in order to promote fairness (Recommendation 2). While the non-specific nature of including some safeguards was important to several participants, it was precisely this lack of specificity – not knowing what was being safeguarded or how – that led to some abstentions. Other participants took issue with the inclusion of “fairness”. For example, one participant felt that this implied that there should be a change to the (perceived) fairness inherent in the current system, while another preferred the terms “equity” or “justice”.

The reasons underpinning Recommendation 2 frequently centered on mitigating ‘seriously declining health’ for candidates waiting for a well-matched kidney. Most wanted to avoid candidates progressing to the high-priority medically urgent status *because of* waiting for an epitope compatible kidney. They suggested relaxing or even removing this criterion in these instances, resulting in Recommendation 3. Some participants envisioned a scoring system, with declining health given additional weight to fast-track kidney allocation. However, some participants felt strongly that kidneys should go to the best-matched candidate to decrease the chances of kidney rejection and the need for re-transplantation. Unfortunately, this session ran overtime, and this recommendation was finalized and voted on after four participants, who were unable to stay longer, left.

As participants worked to further specify the type of safeguards or flexibility around implementing epitope compatibility, the group became more divided. This began with a lengthy discussion about whether there should be a maximum time limit imposed while waiting for an epitope compatible kidney. While some participants were initially in favor of this, quality of life and the health status of transplant candidates arose as more prominent considerations. As captured in Recommendation 4, candidates’ quality of life was incredibly important to prioritize for some participants, who viewed it as a protection against increasing inequities for those living in rural/remote locations. The majority of participants, however, noted that the subjectivity and difficulty with operationalizing quality of life were problematic. Other reasons for voting against or abstaining were that quality of life was viewed as already being part of the current allocation system, it would further complicate the kidney allocation process, and that the emphasis should be on the long-term benefits that epitope compatibility could achieve. One participant suggested that having too many priorities could mean that nothing ends up getting prioritized, and so quality of life could be a determining factor in allocating ~ 10% of kidneys.

The health status of transplant candidates was a frequent focal point throughout the deliberation, and Recommendation 5 was formed to highlight deteriorating health as a priority to safeguard. Some of those in favor stated that this was part of the existing allocation system, which they wanted to maintain. Other participants explained that declining health was inextricably linked with quality of life, and so they were supporting this notion just as they had with Recommendation 4. One participant in support of this safeguard also wanted to impose limitations (e.g., 10% of donated kidneys) to ensure that epitope compatibility was not supplanted, just as they had suggested in Recommendation 4. Several participants did not support this recommendation because they believed Recommendation 3 had a similar focus but was clearer, deteriorating health was already part of the current system, too many priorities would lead to no priorities, and that there was a serious risk of rejection without ensuring epitope compatible transplants.

Ultimately, participants found greater agreement in giving epitope compatibility high, but not absolute priority in kidney allocation (Recommendation 6). Some participants were not comfortable being any more specific; they saw this as policymakers’/clinical experts’ responsibility to implement their guidance. Others believed that this recommendation clarified to policymakers the value and importance participants placed on adopting epitope compatibility in transplantation decision-making. The one abstention felt this was repetitious of earlier recommendations and inherent in the way epitope compatibility would typically be implemented.

### 3. Transition plan and period

Participants wanted policymakers to take well-planned steps towards including epitope compatibility, rather than implementing it immediately. The first step was to conduct a comprehensive and ongoing education program for the public (Recommendation 7). Some who supported the recommendation said that targeting the public could raise awareness of and perhaps even increase kidney donation. Conversely, some voted against or abstained from this recommendation because they believed the program should be focused on those waiting for a transplant, rather than the public, and patients should be prioritized when resources are limited. Another reason for abstaining was the specification that there “needs to be” the education program; the language was too prescriptive.

The other implementation-related step was ensuring that there was a transition plan and period (Recommendation 8). There was concern about the psychological impact of a sudden shift to epitope-based allocation for candidates near the top of the waiting list in the current system; those who were, therefore, likely to be transplanted soon. The discussion included specific suggestions like a “grandfathering clause” or including a set period of time before the epitope system took effect to allow candidates to get used to the new system. In the end, the group opted to leave it to policymakers to decide on the details of the transition period. Similarly, the group wanted to communicate that a transition plan was needed, but did not specify any further details. Those who abstained wanted to limit/restrict the transition period or felt this lacked clarity. Once again, this session went overtime and three participants were unable to stay long enough to vote.

### 4. Ongoing monitoring and assessment

There was unanimous support for assessing epitope compatibility outcomes at least every five years and communicating these results widely to patients, healthcare professionals, and public, whether successful or not (Recommendation 9). This recommendation was partly about evaluating the change in kidney allocation policy; something the group felt should occur on an ongoing basis, in a rigorous manner. Key outcomes identified for monitoring were longevity of graft functioning and transplantation waiting times. The communication aspect of this recommendation was as much about the importance of ensuring transparency as it was about sharing any potential ‘good news’ stories resulting from epitope-guided allocation.

## Discussion

This was the first-known pubic deliberation to collect perspectives and values from members of the general public on changing the deceased donor kidney allocation criteria, despite the interest in this methodology for resource allocation decisions more than a decade ago [33]. Over five weeks, participants deliberated on whether epitope compatibility should be incorporated in the allocation criteria and other kidney allocation policymaking issues. Although participants had no personal experience with kidney disease, they grasped the importance and complexity of kidney allocation, and how epitope compatibility could effect change. The resulting nine participant-generated recommendations were presented to organ donation and allocation policymakers. Recommendations supported adding epitope compatibility to the existing allocation criteria, but participants called for the inclusion of “safeguards” or “flexibility” around how this is implemented, such as relaxing this criterion for those with “seriously declining” health. Moreover, participants stated that a plan and transition period were needed before implementing epitope compatibility, including an ongoing comprehensive public education program. The group unanimously recommended regular monitoring of outcomes of epitope compatible transplants at least every five years, and specified that this should be publicly shared, regardless of the outcomes.

The recommendation to incorporate epitope compatibility into current kidney allocation schemes signals a preference towards more efficiency-based principles of maximizing transplant outcomes in kidney allocation, rather than the traditional equity or wait time-based system. In fact, the group discussed and decided against making a recommendation on imposing an upper limit on waiting time for a deceased donor kidney. This is in contrast to some other studies [34], including a large survey with the general public, which found that waiting time was more important than post-transplant survival in kidney allocation [35]. Gleaned from the information booklet and expert speaker presentations, the current participants expressed an awareness of the scarcity of organs, the growing numbers of patients waiting for transplantation, and embraced scientific advances like epitope compatibility. There was concern that the shortage of organs could be exacerbated (e.g., through higher rejection/re-transplantation rates) if epitope-based allocation was not used.

This shift towards greater emphasis on utility in allocation policy has been increasingly apparent in other jurisdictions for some time [36]. Some patient and healthcare professional preferences have also been documented for better donor-recipient HLA tissue matching over waiting time in kidney allocation [37, 38]. While our findings come from the general public – a group without vested interests in relation to kidney disease – their input is important for several aforementioned reasons, including ensuring trust is maintained in a system that relies on the public’s willingness to donate.

Several studies have assessed public preferences in organ allocation criteria. Similar to our findings, two focus group studies found that the public wanted to save as many lives as possible by prioritizing medically urgent patients and maximizing transplant success [20, 39]. A key consideration was donor-recipient matching on various medical criteria, including tissue matching. While time on the waiting list was considered important in these studies, it was not the dominant priority. This is also consistent with other focus group [40] and survey [41] studies with the general public that specifically addressed kidney allocation. A recent multi-method systematic review found that the most important criteria for allocating organs from the public’s perspective were post-transplant survival (maximizing benefit), age, and medical urgency (need) [21]. Although there were similarities between these other studies and the current deliberation in terms of the public’s preference for prioritizing donor-recipient matching, the previous research was largely focused on establishing the relative importance of different organ allocation criteria, and not necessarily for kidney allocation.

The public deliberants sent a clear message to policymakers when recommending that safeguards and/or flexibility should be part of including epitope compatibility. Most participants supported relaxing the epitope compatibility criterion for those with “seriously declining health”; primarily, to avoid an increase in medically urgent cases. Furthermore, nearly all participants were in favor of epitope compatibility having “high, but not absolute priority” in guiding allocation decisions. Beyond this, the group struggled to find agreement on the specifics of the priorities or protective strategies. Irving et al. noted similar difficulties amongst focus group participants who were trying to decide which organ allocation criteria should be prioritized [20]. Just like with those focus group participants, some of the public deliberants felt the specific details should be decided by clinical or policy experts.

Although concern for candidates’ quality of life while waiting for transplantation was clearly important to the current participants throughout their discussions, they did not collectively agree on prioritizing this over and above epitope compatibility. The main issues were the subjective nature of quality of life and firm utilitarian views on maximizing transplant outcomes through epitope compatibility. Interestingly, several studies involving people with kidney disease [42] and the general public [20, 34, 43] identified quality of life as a high-priority criterion. These studies frequently asked participants to select the most important allocation criteria or choose which hypothetical patients should receive an organ, whereas the current participants were trying to articulate mitigation strategies – safeguards – in response to changing the allocation scheme. Furthermore, participants here were asked to formulate recommendations, which involves carefully constructing statements that they knew would be shared with policymakers, rather than simply expressing a preference in a survey or focus group.

Despite the complexity of kidney allocation and epitope compatibility, the transcripts, list of recommendations, reasons for voting, and positive reactions from policy panelists demonstrated that members of the public from across Canada understood the key trade-offs and policy implications at stake. This study provides a rare opportunity to ascertain the values and perspectives of a group of informed members of the public on cutting-edge genomic science, and whether this should be incorporated into deceased donor kidney allocation criteria. Although the event was held virtually, this was a diverse, pan-Canadian group who were highly engaged in the deliberation throughout. This was marked by frequent referencing to information sources (booklet, expert speakers), sessions going overtime with low attrition, and the high retention rate across five weeks (91%). This study also brought together patients, clinicians, researchers, bioethicists, and policymakers. As recorded in the transcripts, policymakers from four different organ donation and allocation organizations were impressed with the thoughtfulness and level of understanding achieved by the participants on this complex topic. They stated that the recommendations were helpful in mapping the way forward.

Despite these strengths, the findings from this study are not generalizable beyond this participant group, and may not be representative of the wider Canadian public. Similar to any public deliberation, this group came together to deliberate on this specific topic at a particular point in time; current events or personal experiences may have shaped some discussions. Considerable effort, however, went into ensuring diversity of views and experiences through selection on several socio-demographic variables. Additionally, the core principles and values that underscore the recommendations are likely to be more enduring because they were informed jointly by multiple perspectives and types of reasoning; after considering trade-offs prompted by scenarios, having in-depth discussions from various vantage points, and through the cognitive effort required to generate statements and articulate reasons [44].

A second limitation is that this online public deliberation was shorter than the typical four-day in-person event. This likely impacted the degree of interpersonal engagement amongst participants and the depth of deliberation that could have been achieved with more time. Indeed, both of the deliberation sessions went 30–40 min overtime, and 3–4 participants had to leave before voting on Recommendations 3 and 8. Nevertheless, even if all participants who left had voted against those two recommendations, both would remain supported by the majority. It will be important for future research to determine whether longer online sessions might impact participant selection bias and the quality of deliberation.

We acknowledge that the majority of participants in this study self-identified as White, whereas those of Indigenous, Asian, South Asian, Pacific Island, African/Caribbean, and Hispanic backgrounds are disproportionately affected by end-stage kidney disease and are also more likely to experience inequitable treatment [30, 45–47]. Among the 28% of participants who did not self-identify as White, 18% of these self-identified with one of the ethnicities that have elevated kidney disease risk. Despite efforts to include a greater diversity of participants, the COVID-19 pandemic and the online format may have inadvertently introduced challenges to participation for some individuals. This issue could be explored in subsequent in-person deliberations.

## Conclusions

Members of the Canadian public recommended incorporating epitope compatibility as a high-priority criterion for deceased donor kidney allocation, but were cautious around some aspects of implementation. They suggested flexibility to relax the epitope compatibility criterion to prevent undue increases in medically urgent cases, and a transition period to address concerns of candidates in the current system. There was desire for, but lack of agreement on more specific safeguards, which could be addressed by decision-makers. Ensuring transparency and fairness in organ allocation resulted in a call for an ongoing public education program, and regular monitoring and reporting of epitope-guided transplant outcomes. Collectively, these recommendations can provide guidance to policymakers in consideration of adding epitope compatibility to the deceased donor allocation criteria.

## Supplementary Information

**Additional file 1.**

**Additional file 2.**

**Additional file 3.**

## Data Availability

The data used during the current study are not publicly available in order to protect the confidentiality of participants. Deidentified data may be available from the corresponding author on reasonable request.

## Abbreviation

HLA: Human leukocyte antigen
