# Supplementary material for: Adding epitope compatibility to deceased donor kidney allocation criteria: recommendations from a pan-Canadian online public deliberation
Source: BMC Nephrol. 2023 Jun 9;24:165. doi: 10.1186/s12882-023-03224-z (PMC10255937; doi:10.1186/s12882-023-03224-z)
Supplement: Supplementary file 1 — Additional file 1. [file 12882_2023_3224_MOESM1_ESM.pdf]

Considering new criteria for kidney transplantation

A public deliberation

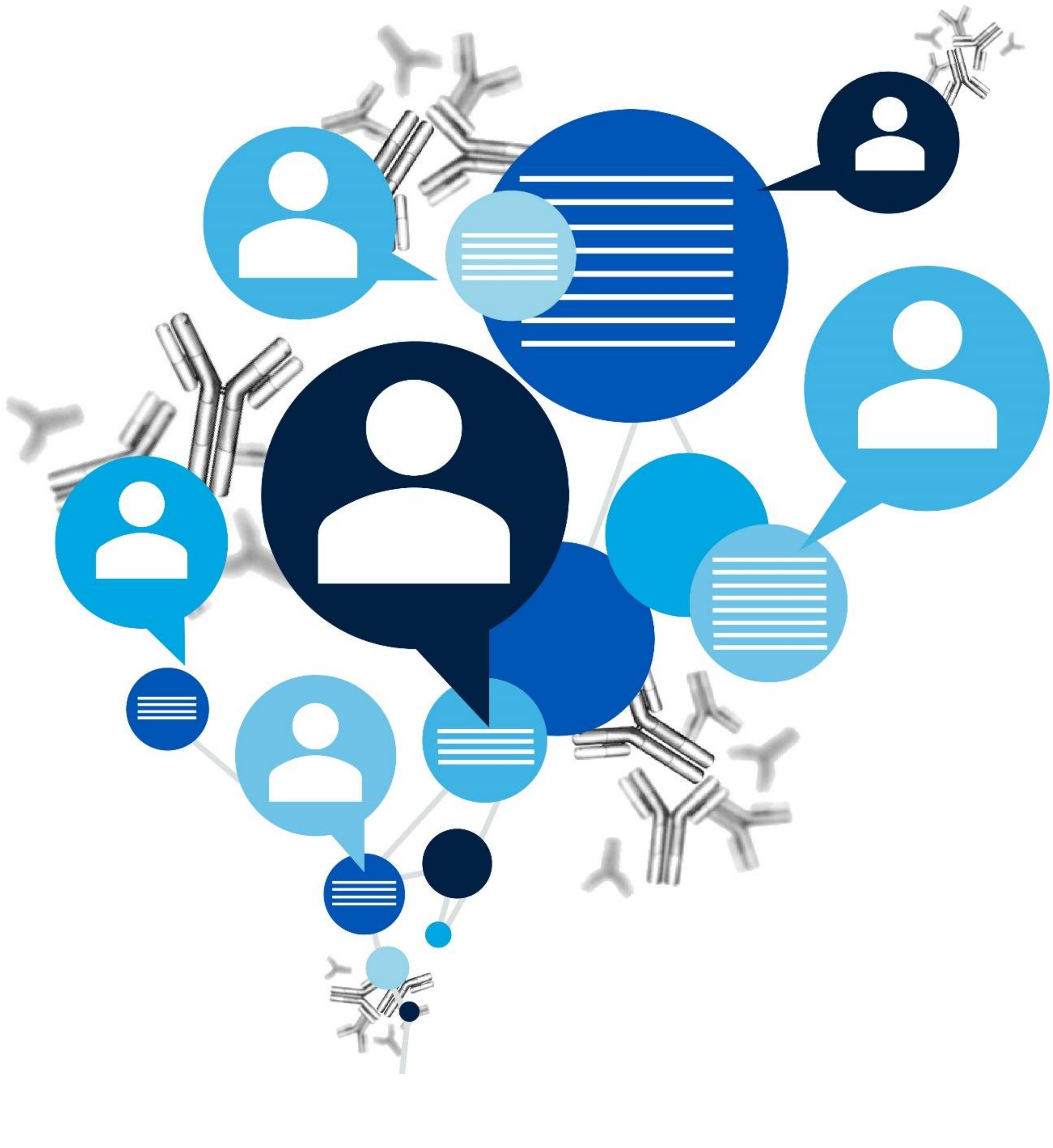

November-December 2021
Virtual event
Hosted by the University of British Columbia

This booklet was prepared by the Kidney Transplant Deliberation Planning Group:

Louisa Edwards
Stirling Bryan
Paul Keown
Ruth Sapir-Pichhadze
Tim Caulfield
Colene Bentley
Michael Burgess
David Hartell
James Lan
Adeera Levin
Marie-Chantal Fortin
Heather Ross
Teresa Atkinson
Jessica Pelletier
Jennifer Desjardins
Austin Kinsella
Mary Bunka

We are grateful to the BC Data Deliberation team for providing content for the introductory public deliberation sections of this booklet and some of the images.

Cover graphic adapted from https://iteachu.uaf.edu/online-discussions/, by Unknown Creator, licensed under CC BY-SA-NC, and antibody image from http://bytesizebio.net/2009/04/10/multitasking-antibody/, by AJC1, Flickr, licensed under CC BY-SA.

The Kidney Transplant Deliberation is part of a larger, multi-component 4-year program of research called 'Precision Medicine CanPREVENT AMR' (https://www.gctransplant.ca/).

This work is being developed and delivered by researchers at the following institutions:

UBC THE UNIVERSITY OF BRITISH COLUMBIA

Centre universitaire de santé McGill / McGill University Health Centre

UNIVERSITY OF ALBERTA

This research is funded by the following non-profit organizations:

GenomeCanada

Genome BritishColumbia

CIHR Canadian Institutes of Health Research / IRSC Instituts de recherche en santé du Canada

Kidney transplant deliberation

1. Purpose of this booklet 2
2. What is a public deliberation? 3
3. Why is this public deliberation important? 4
4. What happens during an online public deliberation? 5
5. End-stage kidney disease 7
6. How is end-stage kidney disease treated? 8
7. Deceased donor wait list and allocation criteria – Standard priority 10
8. High priority deceased donor kidney allocation 11
9. Kidney rejection 12
10. What is epitope compatibility? 13
11. Potential benefits of epitope compatibility 14
12. How would epitope compatibility change the kidney allocation criteria? 15
13. Potential concerns with epitope compatibility 16
14. Summary 17
15. Your role in the deliberation 18
Glossary of terms 19
Additional resources 21
References 23

1. Purpose of this booklet

This booklet is a reference for you to use before and during the public deliberation on matching kidney donors and candidates for kidney transplantation. It covers:

- What to expect as a participant in a public deliberation;
- What end-stage kidney disease is and what treatments are available for it;
- The current systems for matching kidneys and patients in need of kidney transplantation; and
- An introduction to "epitope compatibility" and how it could be used in kidney transplantation decision-making.

Epitope compatibility involves comparing specific immune system molecules between kidney donors and recipients.

The information in this booklet comes from academic literature, ongoing research, kidney organization websites, the media, such as newspapers, and discussions with experts, patients, and stakeholders. Key references are provided at the end of the booklet.

This booklet is meant to give you an overview of the 'Kidney Transplant Deliberation' and help you in your own and the group deliberations about what factors and values are important to consider when it comes to kidney transplantation. You are not expected to be an expert on organ donation, kidney transplantation, medicine, or health policy. Rather, we encourage you to draw on your own experiences, bring your perspectives, your thoughts, and any questions you may have to the deliberation.

You will probably think of other issues or have some questions after reading this booklet. Make a note of these. There will be opportunities for you to ask questions.

There is a glossary of key terms and acronyms at the end of this booklet. These terms appear in **bold** throughout the booklet. A list of resources, such as websites and kidney organizations, is also provided at the end of the booklet.

Please feel free to share this booklet or discuss its contents with other people.

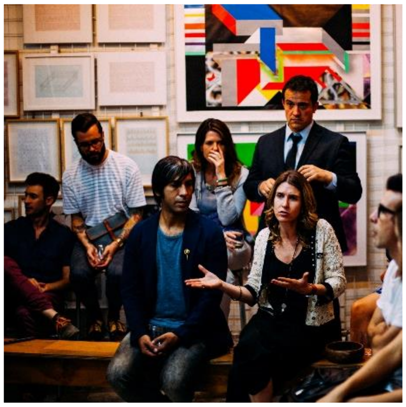
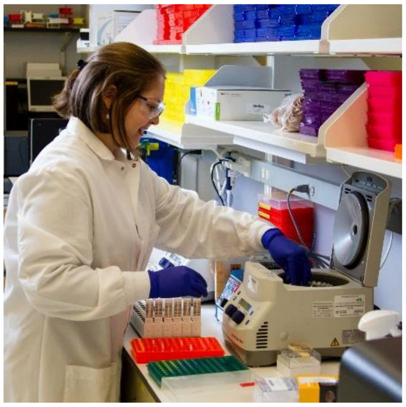
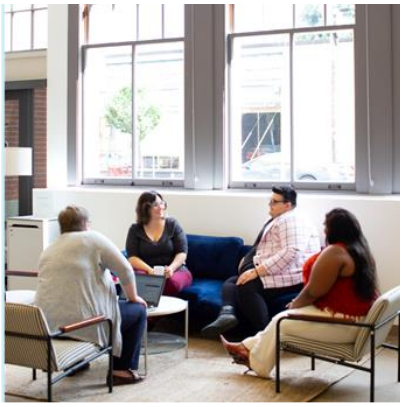
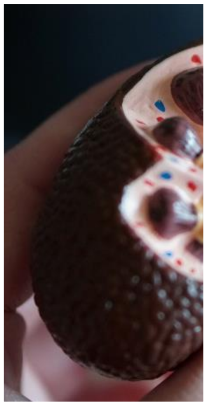

2. What is a public deliberation?

A **public deliberation** is a discussion with members of the public about issues that may have an important impact on our society. Public deliberations are often used to help resolve social dilemmas when evidence is uncertain and value judgements may conflict. Deliberations provide an opportunity for ordinary citizens to be actively involved in collective decision-making.

Public deliberations highlight the potential social and ethical implications of a policy decision for diverse members of the population. These policy decisions are based on values or making trade-offs.

Public deliberations recognize that individuals have different needs, views, and values on complex topics like health care. People invited to participate in a public deliberation are selected to include a range of life experiences that reflect these differences.

> *We encourage you to bring questions to the deliberation, as well as any insights and perspectives you may have.*

Participants are supported in their discussions by trained facilitators, information from expert speakers, an information booklet like this one, as well as the opportunity to engage with their fellow participants. Policymakers, specialists, people with lived experience and others with an interest in the topic may attend a deliberation to provide information or observe the event.

The goal of a public deliberation is for participants to come together as a group and make recommendations on what they collectively consider the best course of action to take on a particular issue, or how we can live together and respect the differences amongst us. Recommendations are voted on by participants, and points of disagreement are noted.

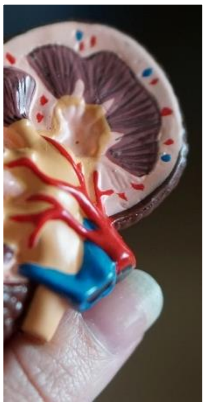
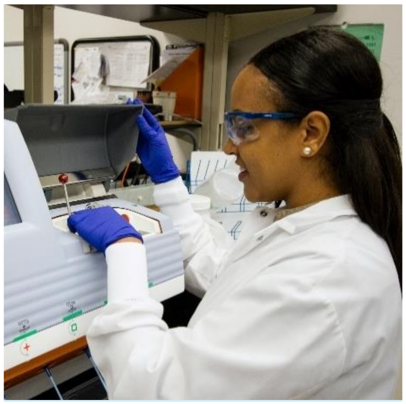
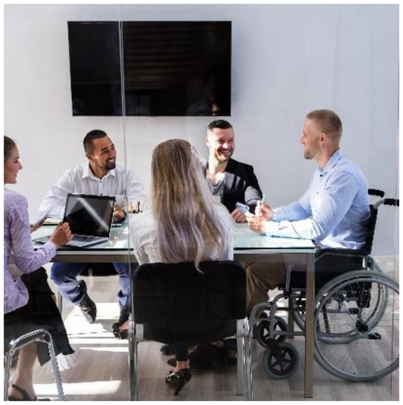
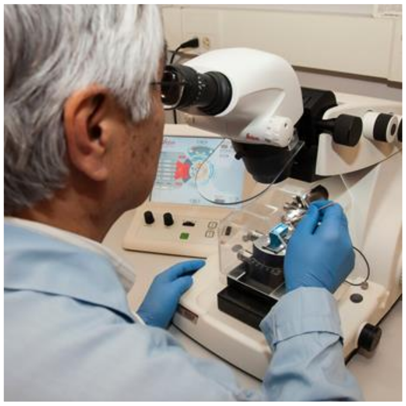

3. Why is this public deliberation important?

There are more patients needing a **kidney transplant** than there are kidneys available for transplantation. This shortage of kidneys means that patients with kidney disease often wait a long time for a kidney to become available to them. Adding to the scarcity issue is that many patients will require more than one transplant over their lifetime, often due to kidney rejection (see Sections 6, 9, 11). Kidney rejection happens when the **immune system** recognizes the transplanted kidney as foreign and attacks it (see Section 9).

A new system for matching kidney donors and recipients that considers **"epitope compatibility"** could reduce this kind of rejection, but it will change the order in which people are offered kidneys and how long they have to wait for one. In this deliberation, the trade-offs we will discuss involve how best to allocate kidneys in a way that is fair to all candidates on the wait list, while balancing how long and how well a transplanted kidney will likely function for the recipient.

People involved in the deliberation will hold a variety of views, and it is important to listen and respect these different opinions. The information you read or hear may shape your opinions, and some of your opinions might (or might not) change throughout the deliberation.

We are inviting members of the public to identify what is important to them about epitope compatibility – a new way of potentially matching donors and recipients for **kidney transplantation** – and to provide suggestions or recommendations to policymakers about this issue. Epitope compatibility is described in Section 10 of this booklet.

It is important to have public input on changing how kidney donors and patients are matched because:

- **kidney failure** is common,
- nearly anyone can choose to become a **deceased kidney donor**, and
- there will be potential benefits and risks to changing the **kidney allocation** and matching system.

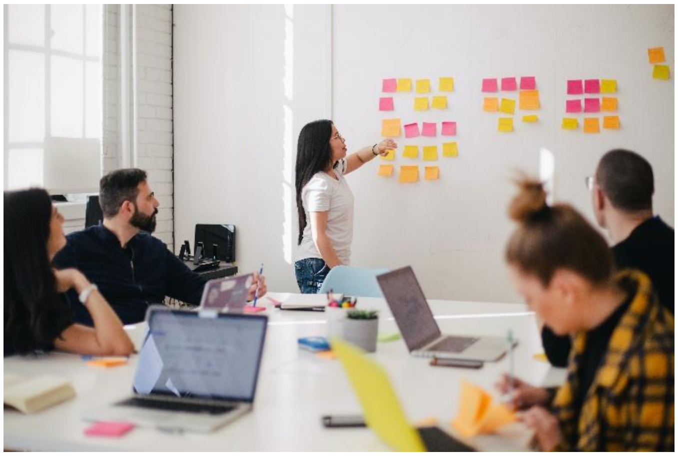

We will discuss how best to allocate kidneys in a way that is fair to all candidates on the wait list, while balancing how long and how well a transplanted kidney will likely function for the recipient.

4. What happens during an online public deliberation?

Public deliberations are usually held in person, but due to the current pandemic, we will be holding this event virtually over Zoom. There will be five sessions, each lasting two hours, held over several weeks. Around 28 people from across Canada will take part in this event.

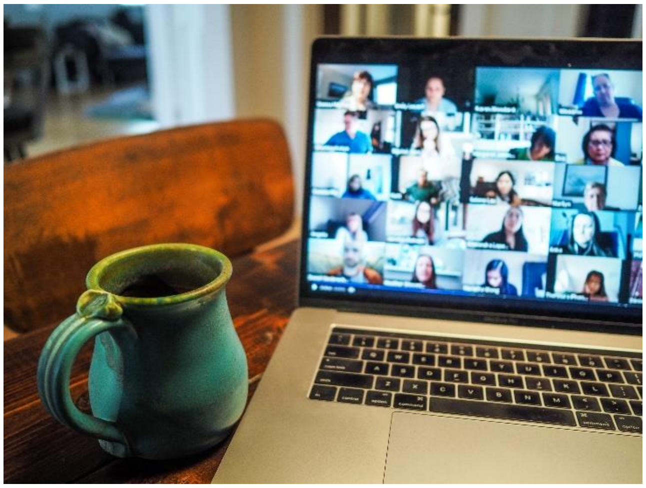

Session 1

Experts will present information about kidney disease, transplantation, and **epitope compatibility**. The speakers are chosen to provide a range of perspectives and may not agree with each other. You will be able to ask them questions to clarify what they have said, and to get more information. All participants will attend this session.

Session 2

In small groups of 6-8 participants, you will work with a facilitator to go through some of the goals of the deliberation. The small group format means everyone will have a chance to share their thoughts. The goal of Session 2 is to identify the different perspectives, beliefs, and values of participants.

Sessions 3 & 4

These sessions will be held with the full group of participants. The group will work with a facilitator to discuss questions that the organizers have prepared. For each question, participants will first discuss their different perspectives on the question and their reasons for them. The purpose of the final two large group sessions is for participants to work together to make recommendations to inform policies on epitope-based kidney allocation.

Session 5

The final session is a policy panel discussion with kidney donation and allocation policymakers from BC Transplant, Trillium Gift of Life, Transplant Quebec, and Canadian Blood Services. Policymakers will review the list of recommendations from Sessions 3 and 4, and it is an opportunity for them to ask the group for more information.

Online Public Deliberation Format

<table>
<tr><th>Online</th><th>Duration</th><th>Who</th><th>Focus</th></tr>
<tr><td>Session 1</td><td>2 hours</td><td>Everyone
(28 participants)</td><td>• Expert speaker presentations
• Ask questions and get more information</td></tr>
<tr><td>Session 2</td><td>2 hours</td><td>Small group
(6-8 participants)</td><td>• Get to know other participants
• Review the goals of the deliberation
• Consider a broad range of perspectives</td></tr>
<tr><td>Session 3</td><td>2 hours</td><td>Everyone
(28 participants)</td><td rowspan="2">• Discuss deliberation questions:
○ Explore different beliefs and the reasons for them
○ Suggest & vote on policy recommendations
• Review all recommendations and revise, if needed</td></tr>
<tr><td>Session 4</td><td>2 hours</td><td>Everyone
(28 participants)</td></tr>
<tr><td>Session 5</td><td>2 hours</td><td>Policy Panel (4) +
Everyone
(28 participants)</td><td>• Review & discuss recommendations
• Ask participants for clarification</td></tr>
</table>

Example deliberation questions

Under what conditions is it appropriate to use epitope-based compatibility?

What limits, if any, should be considered when introducing epitope-based compatibility?

5. End-stage kidney disease

Kidneys are a pair of vital organs that purify our blood by eliminating waste products, toxins and excess fluid from the body. Kidney disease usually attacks the tiny filters in the kidneys, making it harder for them to remove the waste products and excess fluid. If this damage occurs quickly it is known as **acute kidney disease**. If it lasts for three months or longer, a person is said to have **chronic kidney disease**.

Kidney disease was the 10th leading cause of death in Canada in 2019. It can affect people of any age. In 2019, 46% of patients newly diagnosed with kidney disease were under the age of 65. Symptoms include weight loss, fatigue, weakness, nausea/vomiting, shortness of breath, chest pain, leg cramps, restless legs, easy bruising, swelling in the legs/ankles, to name a few.

Chronic kidney disease is common, with 1 in 10 Canadians – more than 4 million people – diagnosed with this condition. It can range from mild to severe in nature, but chronic kidney disease may progress to **kidney failure**, which is also known as **end-stage kidney disease**. In this stage, the kidneys no longer work well enough to remove toxins and excess fluid from the body.

Diabetes is the leading cause of kidney failure. Having high blood pressure or a family history of kidney disease also increases the risk of developing kidney disease. Other people may have a genetic condition that causes kidney failure. In addition, people of Indigenous, Asian, South Asian, Pacific Island, African/Caribbean and Hispanic ethnic backgrounds are at increased risk of kidney disease, although it affects people of all backgrounds.

In Canada, the number of people with end-stage kidney disease has increased by 35% since 2009, partly due to people living longer and increases in diabetes and high blood pressure.

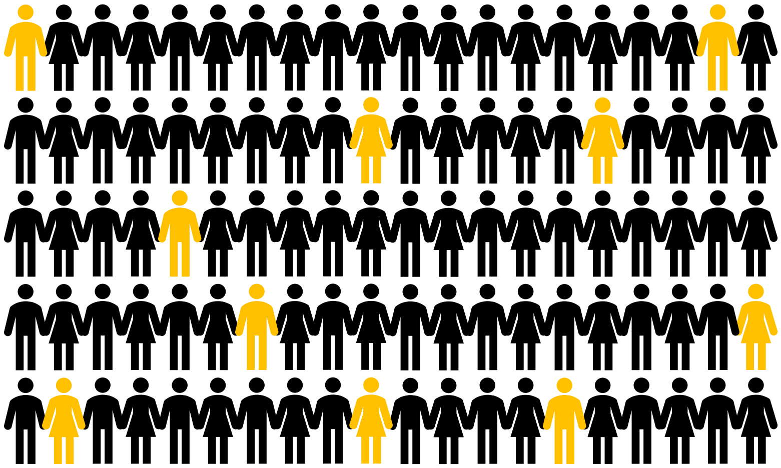

1 in 10 Canadians have chronic kidney disease

6. How is end-stage kidney disease treated?

After progressing to **end-stage kidney disease**, kidney replacement therapy is necessary to stay alive. The treatment options are **dialysis** (58% of end-stage kidney patients in Canada) and **kidney transplantation** (42% of end-stage kidney patients). People with end-stage kidney disease will usually need to remain on dialysis for the rest of their lives, unless they have a successful kidney transplant.

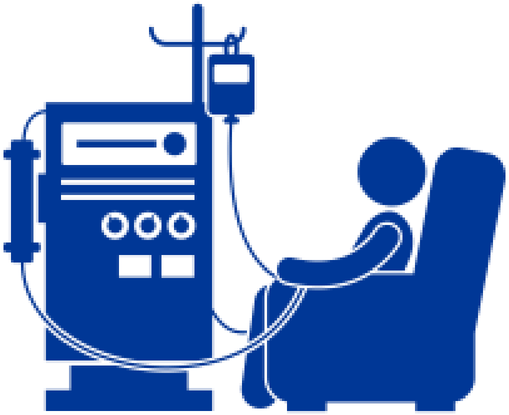

A kidney transplant is the preferred treatment for end-stage kidney disease because it greatly improves survival and quality of life for the patient. Transplantation also decreases annual treatment costs by 50-70% compared with facility-based dialysis ($25,000 vs $50,000-$83,000/year).

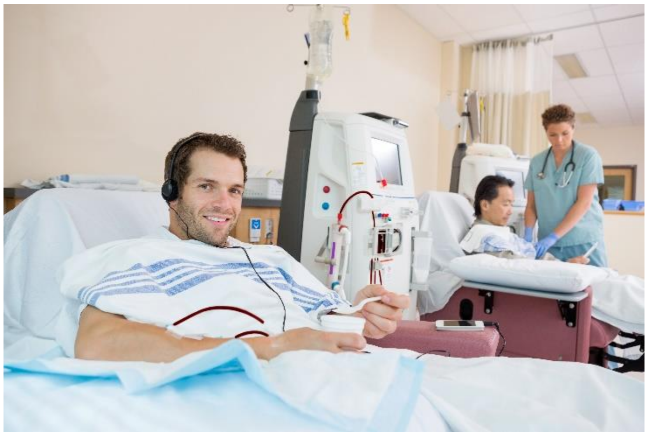

Dialysis

Dialysis is a treatment that mechanically cleans the blood and removes excess fluid when the kidneys are no longer working well enough to do this. It is available at home or in a hospital or clinic, but more than 75% of dialysis patients in Canada get treatment in a hospital or clinic.

With clinic- or hospital-based dialysis, the patient must travel to the clinic for treatment three times per week, for about four hours each time. Blood is withdrawn through a needle in the arm and passes through a dialysis machine where the toxins and excess fluid are removed. The filtered blood is then returned to the patient’s body. To manage the build-up of wastes and fluids between treatments, dialysis patients have a daily fluid limit and a restricted diet.

Clinic-based dialysis

3 visits per week

4 hours per visit

Kidney transplantation

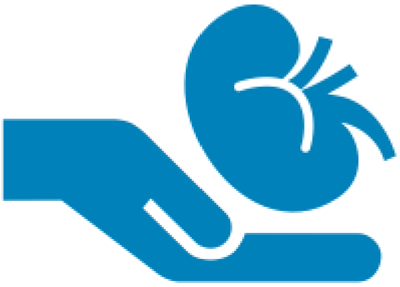

Having a **kidney transplant** is major surgery and it's not an option for everyone with kidney failure. Patients must have a thorough medical assessment to confirm they are healthy enough to have the surgery. They also need to be able and willing to follow a treatment and medication plan after surgery. The assessment can take several months to complete.

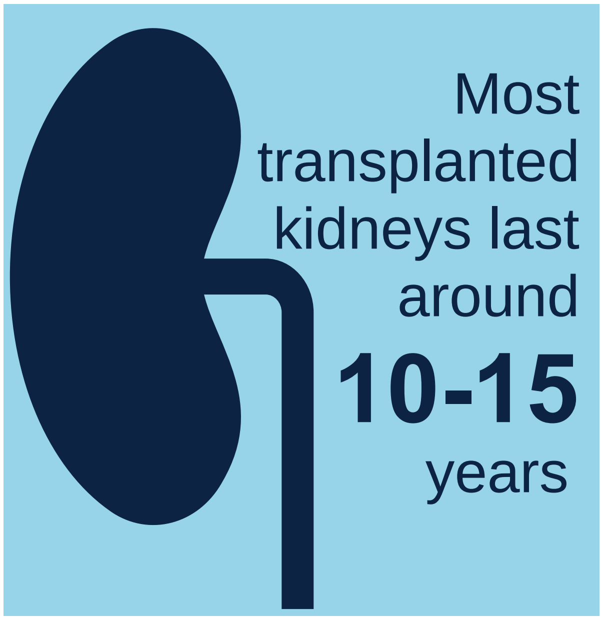

If a patient is a suitable candidate for a kidney transplant, surgery is scheduled when a compatible kidney is available. Blood type must be compatible between the donor and recipient, and the kidney must be healthy and functioning. Another test checks that the candidate's **immune system** does not contain protein molecules that would attack the donor kidney.

There are currently two sources of kidneys available to transplant candidates – **living kidney donors** and **deceased kidney donors**. A living kidney donor is a family member, friend or any other person with a compatible kidney who is willing to donate it to a transplant candidate. If there is no suitable living donor, the candidate is placed on a waiting list and a kidney can come from someone who has died, called a **deceased donor**.

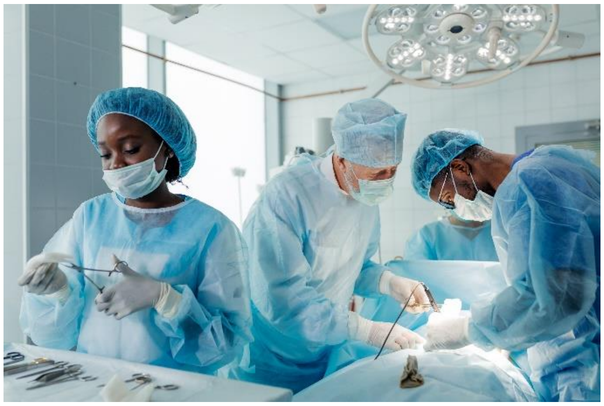

After transplant surgery, recipients must take **immunosuppressant** medications every day to prevent kidney rejection. Side effects from these medications can be quite unpleasant. They include stomach irritation, trouble sleeping, weight gain, swelling of the hands, face, or ankles. They can even lead to other serious health conditions, such as cancer or heart disease.

If the recipient follows their treatment plan and there are no post-transplant complications, a transplanted kidney may function well for the recipient's lifetime. On average, transplanted kidneys continue to function for around 10-15 years.

7. Deceased donor wait list and allocation criteria – Standard priority

A patient waiting for a kidney from a **deceased donor** must register on the deceased donor waiting list in their region, which is usually the province they live in or closest to. Donated kidneys are not normally shared between regions, except for high-priority patients, as described below in Section 8.

Wait times

There are more people who need a transplant than there are kidneys available from deceased donors. The number of candidates needing a **kidney transplant** also increases each year. The longer candidates remain on **dialysis** waiting for a transplant, the greater their chances of becoming more ill or dying.

For example, around 3% of candidates in BC – 11 people – die every year while waiting for a transplant, and wait times in BC are among the shortest in Canada.

The average amount of time that adults are on the wait list in Canada is 4 years, but it varies depending on where the candidate lives. The shortest wait time is in Nova Scotia (3 years) and the longest is in Manitoba (6 years).

The range of wait times across Canada is partly determined by the population density differences, the number of donors, and the characteristics of people on the wait list. For example, it can take much longer to find a compatible kidney for candidates with less common blood group types, such as blood type B.

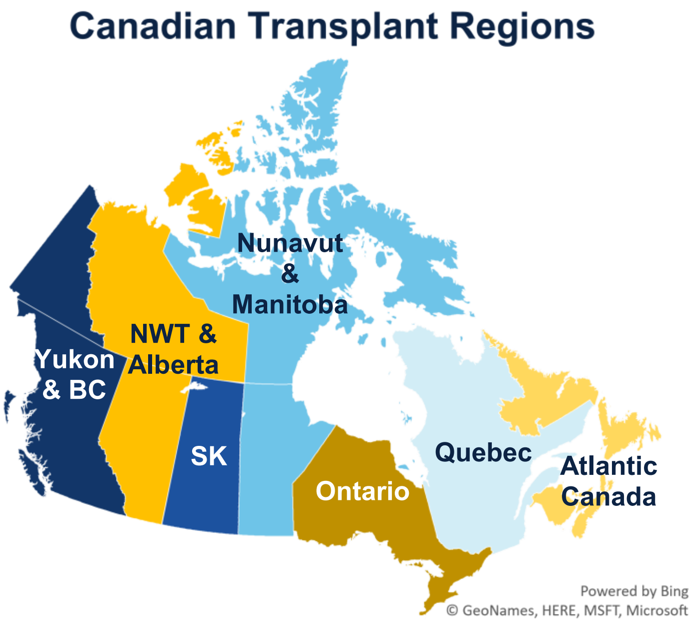

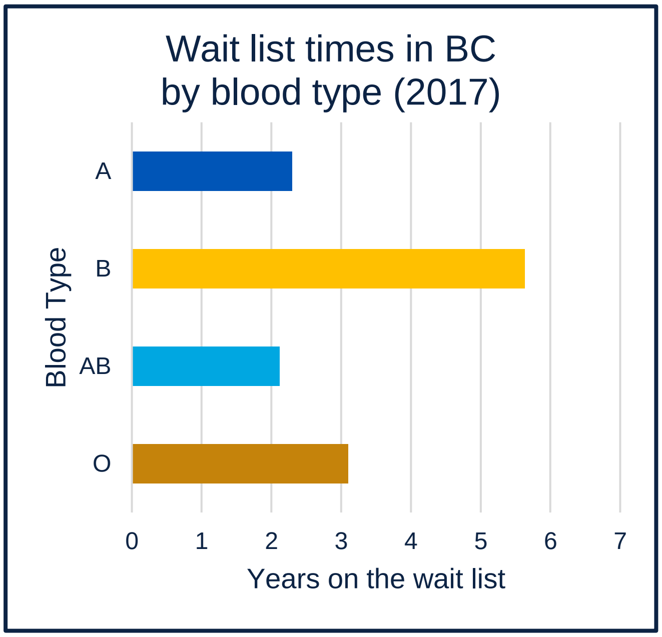

Allocation criteria

Kidney allocation is the process of deciding who should be offered the next available kidney from a deceased donor. Decision rules are needed because kidneys are a scarce resource; there are more patients who need a transplant than there are kidneys available for transplantation.

Current kidney **allocation criteria** in Canada already considers some matching and compatibility between donors and recipients. For example, an effort is made to match the age of donors and recipients. The blood group of donors and recipients must also be compatible. Another blood test checks that the recipient does not have **immune system** proteins (called antibodies) that will reject the donor kidney.

Kidney allocation is complex, and each transplant region has slightly different rules to determine the order in which candidates are offered a kidney. In general, current kidney allocation policy and practice in Canada assigns the next available deceased kidney to the next compatible candidate on the waiting list, but does take into account time on **dialysis** and other clinical factors.

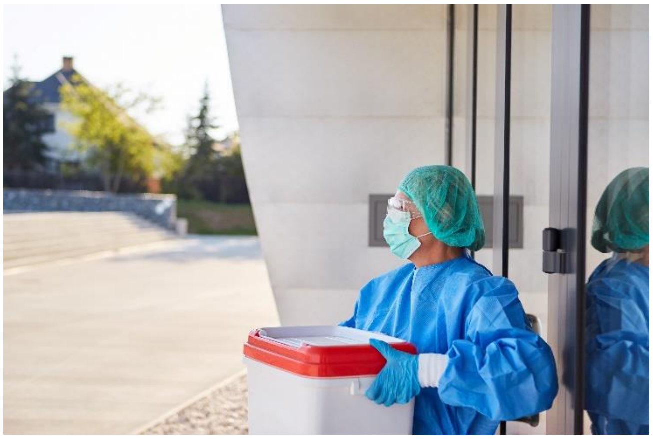

8. High priority deceased donor kidney allocation

Some transplant candidates are considered higher priority, which means they are offered a suitable kidney ahead of others on the waiting list. These patients are children, have a medical urgency (e.g., unable to access veins for dialysis), or are considered "highly sensitized".

Highly sensitized patients have antibodies against 95% or more of the population, and so wait much longer for a kidney, which increases their chance of becoming more ill or dying while they wait. In order to help these hard-to-match candidates, the Highly Sensitized Patient Program was created in Canada. When a pair of kidneys is received from a deceased donor, one kidney is offered to the national Highly Sensitized Patient Program and the other kidney remains within the province/territory for allocation according to that region's **allocation system**. Therefore, highly sensitized patients have access to a larger, national pool of kidneys, which increases their chances of finding a suitable match.

9. Kidney rejection

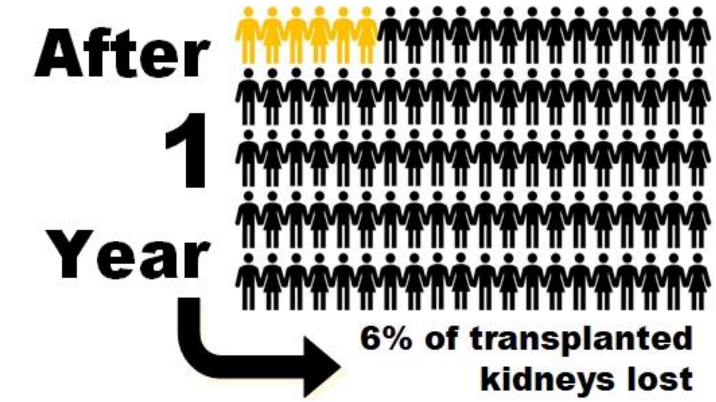

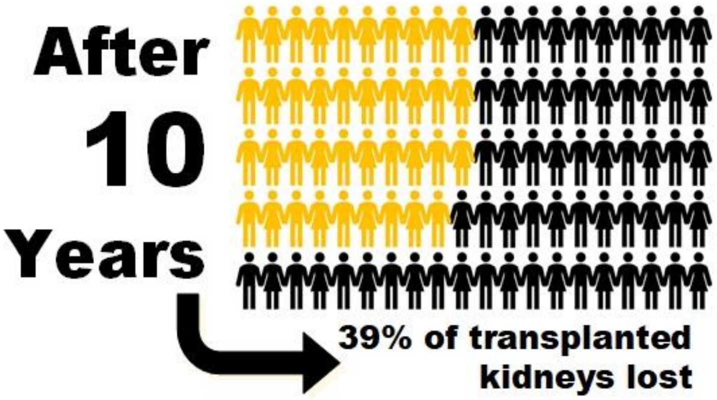

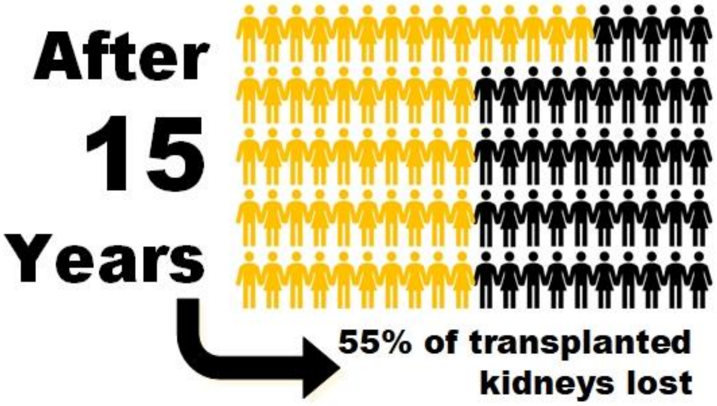

Canada's **kidney transplant** success rate is amongst the best in the world, with 94% of deceased donor transplanted kidneys working for at least the first year. Unfortunately, more than one-third of transplanted kidneys are lost by 10 years and more than half by 15 years post-transplant. While many factors may jeopardize the transplanted organ, the overwhelming cause of **graft** loss (when the transplanted kidney, or graft, no longer functions properly) is rejection.

Antibody-mediated rejection remains the most serious and destructive form of rejection. It may occur early or late after transplantation and happens when the body recognizes the transplanted kidney as foreign and immune cells attack the kidney. One of the most common causes of rejection is skipping doses of immunosuppressants or not taking them in the way they were prescribed. This may be caused by illness or unpleasant side effects from the medication. A lower dose of medication allows the **immune system** to become stronger and create antibodies against the transplanted kidney. This process damages the transplanted kidney.

Antibody-mediated rejection is now a major cause of losing a transplanted kidney, and there are virtually no effective therapies to reverse this process once it has begun. In time, the transplanted kidney will no longer function properly. The recipient must then go back on dialysis until another kidney becomes available for transplant. However, rejection can result in a recipient becoming highly sensitized. It would then be harder to find a compatible kidney donor for any future transplants. It also means that the candidate will be competing for the same scarce supply of kidneys again. Every re-transplant surgery means fewer kidneys are available for other candidates.

10. What is epitope compatibility?

The **immune system** detects and destroys foreign substances in the body to protect it from illness. A foreign substance is called an **antigen**. Immune cells produce a protein, called an **antibody**, in response to an antigen.

Antibodies are designed to detect specific antigens. When an antibody gets close to its antigen, they bind together. The area of the antigen (foreign substance) that sticks to the antibody (immune system protein) is called the **epitope**.

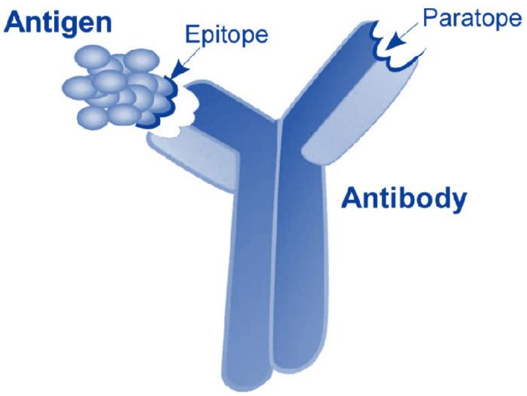

An epitope is the area of the antigen that sticks to the antibody (Y-shaped immune system protein).

The immune system uses **human leukocyte antigens (HLAs)** to distinguish self from non-self. Some transplant centres try to match HLA antigens between donors and recipients to avoid kidney rejection. If the antigens are the same, antibodies will not be produced against the transplanted kidney. It's very difficult to match HLA antigens because there are tens of thousands of them.

While HLA matching may not be feasible, recent advances in research mean it may be possible to match some epitopes. Epitope matching is easier to carry out because there are hundreds of epitopes, rather than thousands.

Not all epitope mismatches cause antibodies to develop. **Epitope compatibility** considers specific epitope matches and mismatches between donors and recipients. The degree of epitope compatibility is determined by the number of mismatches between the donor and recipient.

For this deliberation, epitope compatibility is only being considered for deceased donor kidney transplants, which tend to have poorer outcomes than living donor transplants. Research is currently underway to identify which epitopes are the most important to match between kidney donors and recipients, and to understand the extent to which epitope compatibility could be achieved successfully in Canada.

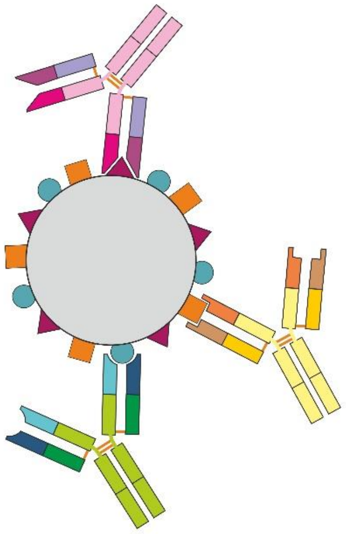

Y-shaped antibodies bind to specific epitopes (circles, squares, and triangles) on the surface of an antigen (grey circle).

11. Potential benefits of epitope compatibility

Better **epitope compatibility** between a donor and recipient leads to a longer-lasting, better functioning kidney. Several studies have shown that epitope compatibility reduces the risk of rejection and transplanted kidney (**graft**) failure. This is clearly beneficial to the health and quality of life of all transplant recipients. Having a longer-lasting, better functioning kidney may be particularly helpful for younger patients. Younger patients often need more than one transplant over the course of their life. With epitope compatibility, it is possible that a transplanted kidney could last for a recipient's lifetime. This means that fewer recipients would go through the difficult experience of kidney rejection and re-transplantation.

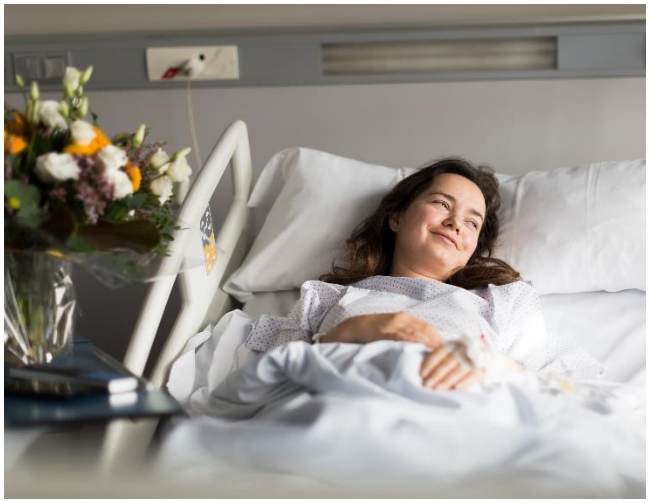

Improving kidney functioning and survival through epitope compatibility will lead to long-term benefits to the health system. If the rate of graft loss due to rejection is decreased, fewer recipients will need more than one transplant. Over time, this would result in a greater supply of deceased donor kidneys available for other transplant candidates. Fewer transplants per recipient means that more candidates could receive a kidney transplant. There is also a long-term economic benefit to the health system of improving transplantation outcomes. Yearly treatment costs for dialysis (around $50,000-$83,000/year) are greater than the medication costs for transplant recipients (around $25,000/year).

A donated kidney should be transplanted as soon as possible after removal from the donor because this gap affects the long-term outcome for the recipient; how well the kidney works and its survival decreases as the time between donation and transplantation increases. Early results show that it takes up to 6 hours for epitope compatibility testing. This is within the 12-hour timeframe used to match a kidney with a recipient in the current system. Therefore, it appears that epitope compatibility testing can be done without adversely affecting the kidney.

12. How would epitope compatibility change the kidney allocation criteria?

Canada currently prioritizes *time spent on the waiting list* when determining who should be offered the next available deceased donor kidney (see Section 7). Including **epitope compatibility** in **kidney allocation** would prioritize the *outcome* of transplantation. This means allocating kidneys to maximize recipient and **graft** survival. A donated kidney would be offered to the recipient who is most epitope-compatible with the donor.

Time on the waiting list would be a less important consideration under epitope compatibility than it is now. However, it could still play an important role in decision-making. For example, if two candidates are equally well matched with a donor kidney, time on the waiting list could break the tie between them.

Adding epitope compatibility to the **allocation criteria** would be decided by each transplant region in Canada. A policy change to the allocation criteria would apply to all standard priority kidney transplant candidates in a transplant region. This means that individuals could not choose to opt in or out of this allocation scheme, if it is adopted in their region.

Epitope compatibility would not change the priority given to the groups listed in Section 8, such as children or those with a medical urgency. Candidates are likely to fall into two broad groups that epitope compatibility would impact: those that are easy to match and those that are more difficult to match. Candidates with common blood types (e.g., A) and little or no pre-existing antibodies are currently easier to match. This might change by introducing epitope compatibility testing. For example, waiting times are mostly likely to increase for those with little or no pre-existing antibodies and those with blood group B.

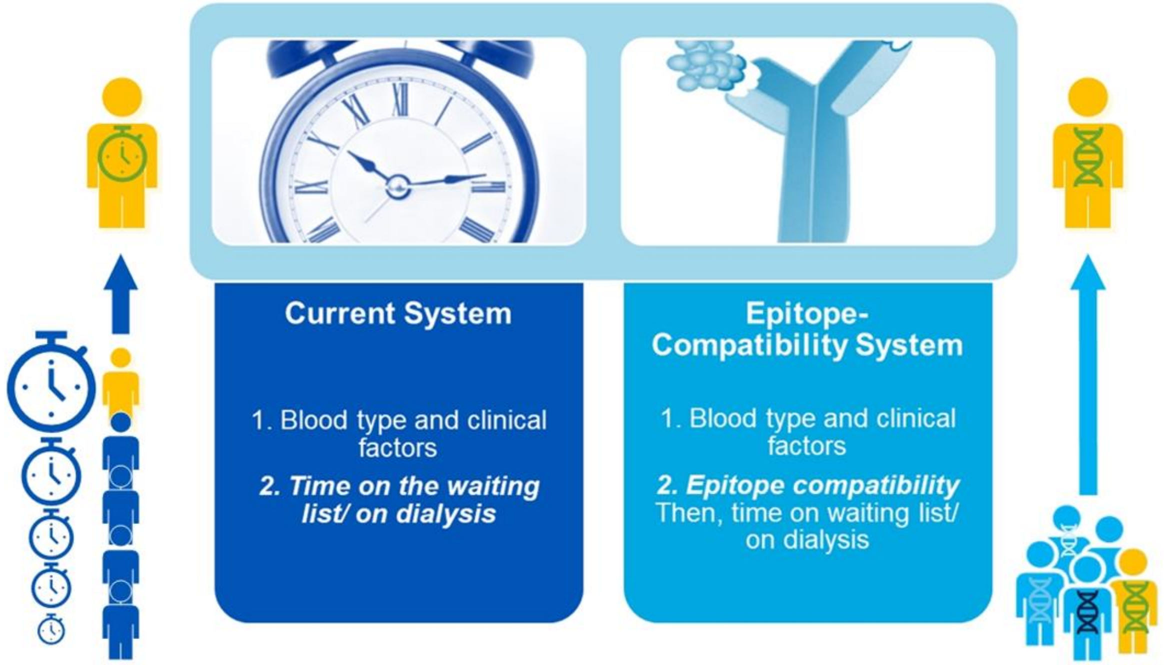

13. Potential concerns with epitope compatibility

It is possible that some candidates on the wait list may be disadvantaged by using **epitope compatibility** to guide **kidney allocation** decisions. First, changing the kidney allocation system to include epitope compatibility could mean that some people who have already been on the wait list for several years might have to wait even longer for a compatible match. Under the current allocation system, the longer candidates are on the wait list, the closer they are to receiving a kidney. However, this may no longer be the case if the system is based on epitope compatibility. Considering what is fair to candidates who have already waited a long time under the current system is important.

Second, some candidates may be missing epitopes that are common in many donors. These candidates could wait an exceptionally long time for a well-matched kidney. A longer wait would increase their chances of becoming more ill or dying while waiting for an epitope-compatible kidney. Introducing safeguards could prevent these situations, but these recipients might not benefit from epitope compatibility.

Third, using epitope compatibility to guide allocation decisions will lead to more uncertainty over how long candidates must wait for a transplant. Candidates already experience some uncertainty over waiting times, which can be a source of considerable stress. Epitope compatibility is mostly likely to increase this uncertainty for groups of candidates that are already challenging to match because of their blood type. There is, by definition, a smaller number of available donors for those who are more difficult to match.

Epitope compatibility would be added to the existing list of clinical factors that are matched between donors and candidates. Candidates with the same clinical factors would be in a large group or pool, and they would all be considered each time a kidney becomes available. The time candidates wait for a transplant will depend on how frequently donors have epitopes similar to their own. This will make it harder to give an accurate prediction about waiting times.

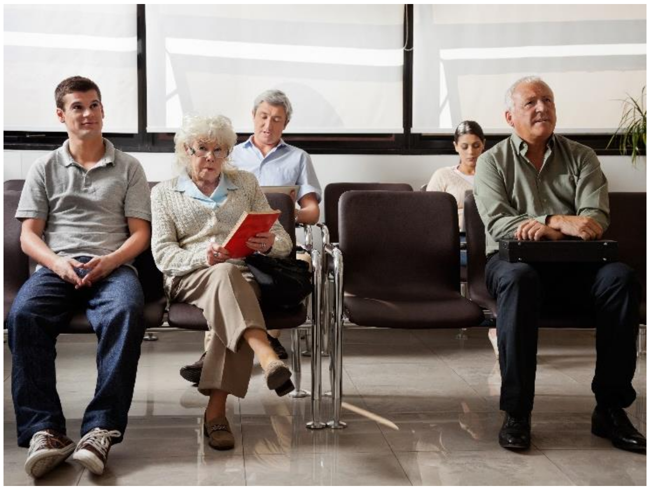

14. Summary

Due to an aging population and more people with diabetes, the need for kidney transplants is steadily increasing. The number of donor kidneys, however, is limited. Reducing rejection and improving transplantation outcomes are key priorities for the health system. Better kidney transplant outcomes will improve the health and quality of life of transplant recipients. One approach being considered is using epitope compatibility to guide transplantation decisions, which may provide exactly these kinds of benefits.

Improving transplantation outcomes through epitope compatibility could, at the same time, introduce other issues. Transplant candidates who are missing common epitopes may wait longer than under the current allocation system. Uncertainty around wait times for transplantation could be increased, especially for those groups of candidates that are already more difficult to match to donors.

It is, therefore, important to develop policies and regulations that can balance the benefits of improved transplantation and the risks of changing the deceased donor kidney allocation criteria.

15. Your role in the deliberation

During the deliberation, you will hear more from experts about **end-stage kidney disease**, its treatment, and how **epitope compatibility** could change transplantation decision-making.

You and your fellow deliberants will bring your own perspectives to the discussion. You are not expected to be an expert on this topic.

As part of a group, you will be asked to discuss some of the issues related to using epitope compatibility in kidney allocation decisions.

These may include questions such as:

- *Under what conditions is it appropriate to use epitope-based compatibility?*
- *What limits, if any, should be considered when introducing epitope-based compatibility?*

We hope that you will bring your opinions, values, and ideas about **kidney transplantation** decision-making to the deliberation. You will work together with the other participants to make recommendations for policy makers in this area. Policy makers will take your recommendations into account as they consider changing the **kidney allocation criteria**.

To help make this discussion as successful as possible, we ask that you:

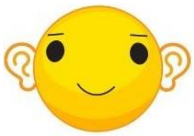

Listen to others

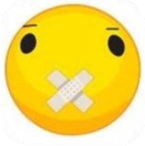

Avoid cross-talk

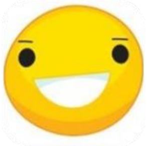

Keep an open mind

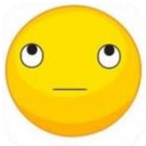

Don’t roll your eyes

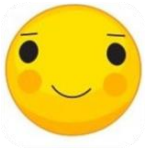

Resist checking email, social media or texting

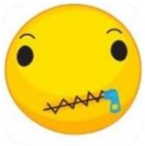

Try not to interrupt

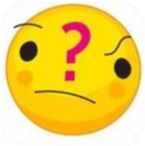

Ask questions

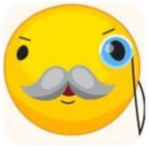

Try to justify your opinions

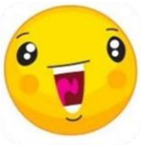

Stay engaged

Glossary of terms

acute kidney disease: a sudden loss of kidney function between 7-90 days after an event that causes the kidneys to suddenly stop working

allocation/allocation system: See *kidney allocation/kidney allocation system*

allocation criteria: See *kidney allocation criteria*

antigen: a toxin or other foreign substance in the body that triggers an immune response, which usually involves producing antibodies

antibodies: blood proteins that are made by immune cells in response to antigens

antibody-mediated rejection: when the body recognizes a transplanted kidney as foreign and creates antibodies that damage the transplanted kidney

chronic kidney disease: a disease that attacks the filtering units of the kidneys and reduces their ability to remove wastes and excess fluids that lasts for three months or longer, and leads to permanent kidney damage

deceased kidney donor: a person who died and had agreed to donate their kidney(s) after their death

dialysis: a treatment that cleans the blood and removes excess fluid from the body when kidneys are no longer working well enough to do this

end-stage kidney disease: the fifth and final stage of chronic kidney disease, when kidneys are no longer functioning well enough on their own (dialysis or transplantation is required); also known as *kidney failure*

epitope: the antibody binding site on an antigen

epitope compatibility: matching specific immune system molecules between kidney donors and candidates; fewer mismatches indicates greater compatibility

graft: a transplanted kidney

highly sensitized patients: people who have HLA antibodies against 95-100% of the population

human leukocyte antigens (HLAs): caused by the body to distinguish itself from foreign substances in the body

immune system: the system that protects the body from illness by detecting and removing foreign substances

immunosuppressant: medication that prevents the body from rejecting a transplanted kidney; they stop the immune system from recognizing the transplanted kidney as foreign

kidneys: a pair of vital organs that eliminate wastes, toxins and excess fluids from the body

kidney allocation/kidney allocation system: the process of deciding who should be offered the next available deceased donor kidney

kidney allocation criteria: information used to make decisions about who should be offered the next available kidney, such as blood type or time on the waiting list

kidney failure: See *end-stage kidney disease*

kidney transplant/kidney transplantation: a surgical procedure to place a healthy kidney from a donor into the body of someone with kidney failure; the new kidney takes over the work of the non-functioning kidney

public deliberation: a discussion with community members as part of the decision-making process for a complex issue

Additional resources

The Kidney Foundation of Canada

Select your region using the button at the top right of the homepage: kidney.ca

"What is Kidney Disease?" https://kidney.ca/Kidney-Health/Newly-Diagnosed/What-is-Kidney-Disease

Kidney PLUGGED IN

Kidney health information videos from The Kidney Foundation, BC & Yukon Branch: www.youtube.com/c/KidneyPLUGGEDIN

"Important facts about kidney disease YOU should know" www.youtube.com/watch?v=KGkM_pIrw6c&list=PL1xafqJq-rs0MdLV1-VUvxKHhW4pb2P0m&index=6

"8 Important things YOU should know about registering to be an Organ Donor!" www.youtube.com/watch?v=L1gRl3xur68&list=PL1xafqJq-rs2cPW3gCB937omX06M0AC1X&index=4

"Did YOU Know? 1 in 10 Canadians has kidney disease. Be informed and learn the early warning signs." www.youtube.com/watch?v=h3lcNKPVZr8&list=PL1xafqJq-rs2cPW3gCB937omX06M0AC1X&index=2

Dialysis

"What is Dialysis?" en.horizonnb.ca/home/facilities-and-services/services/clinical-services/dialysis-(nephrology).aspx

"About dialysis" https://www.ontariorenalnetwork.ca/en/kidney-care-resources/living-with-chronic-kidney-disease/about-dialysis

Epitope compatibility

"Precision medicine and epitope matching in kidney transplantation" www.youtube.com/watch?v=wLiJeJQcrPQ

Kidney Allocation

Manitoba: www.transplantmanitoba.ca/transplant-program/kidney-allocation

Ontario: www.giftoflife.on.ca/resources/pdf/transplant/Kidney_Infographic_final_en.pdf

Québec:
www.transplantquebec.ca/sites/default/files/patientsenattente_aout2012_en_0.pdf

Kidney Health

Kidney Health Check: Early Diagnosis, Videos, Risk Factors www.kidneyhealthcheck.ca

Kidney Health Self-Assessment quiz www.bcrenal.ca/health-info/prevention-public-health/kidney-health-self-assessment

Kidney Transplantation

"About Transplantation" https://www.ontariorenalnetwork.ca/en/kidney-care-resources/living-with-chronic-kidney-disease/about-transplantation

"Introduction to Kidney Transplant: A Step by Step Process" www.youtube.com/watch?v=mKEHbyzjxnU

Transplant statistics for 2016-2020: See "Facts" on the homepage of the Multi-Organ Transplant Program of Atlantic Canada (MOTP) www.motpatlantic.ca

"Focus on Transplantation" giftoflife.on.ca/en/transplant.htm

"Kidney Transplant as a Treatment Option for Chronic Kidney Disease" www.giftoflife.on.ca/resources/pdf/transplant/1_Introduction_to_Kidney_Transplant.pdf

"Surgery Overview" myhealth.alberta.ca/Health/pages/conditions.aspx?hwid=aa94698

Organ Donation

"Why Canada Needs More Organ Donors" https://kidney.ca/KFOC/media/images/PDFs/Facing-the-Facts-2021_Organ-Donation_EN.pdf

References

[Note: In the interests of enhancing readability for non-expert audiences, in-text citations were not included throughout the booklet.]

BC Transplant Kidney Annual Report (KAR). 2017 Renal Program. Internal report of activities and outcomes.

BC Transplant. http://www.transplant.bc.ca/transplant-and-medications/general-medication/rejection

Canadian Blood Services. https://professionaleducation.blood.ca/en/organs-and-tissues/programs-and-services/highly-sensitized-patient-hsp-program

Kidney Foundation of Canada. Facing the Facts. https://kidney.ca/KFOC/media/images/PDFs/Facing-the-Facts-2020.pdf

Kidney Foundation of Canada. https://kidney.ca/Kidney-Health/Living-With-Kidney-Failure/Dialysis

Kidney Foundation of Canada. https://kidney.ca/Kidney-Health/Living-With-Kidney-Failure/Dialysis

Kidney Foundation of Canada. Risk Factors. https://kidney.ca/Kidney-Health/Newly-Diagnosed/Risk-Factors

Organ replacement in Canada: CORR annual statistics, 2019, https://www.cihi.ca/en/organ-replacement-in-canada-corr-annual-statistics-2020

Sapir-Pichhadze R, Zhang X, Ferradji A, et al. Epitopes as characterized by antibody-verified eplet mismatches determine risk of kidney transplant loss. *Kidney Int*. 2020;97:778-785.

Sellares J, de Freitas DG, Mengel M, Reeve J, Einecke G, Sis B, et al. Understanding the causes of kidney transplant failure: the dominant role of antibody-mediated rejection and nonadherence. *Am J Transplant*. 2012;12(2):388-99.

Senev A, Coemans M, Lerut E, et al. Eplet mismatch load and de novo occurrence of donor-specific anti-HLA antibodies, rejection, and graft failure after kidney transplantation: An observational cohort study. J Am Soc Nephrol. 2020;31:2193–2204.

Statistics Canada. Table 13-10-0394-01 Leading causes of death, total population, by age group. https://www150.statcan.gc.ca/t1/tbl1/en/tv.action?pid=1310039401

Tonelli M, Wiebe N, Knoll G, et al. Systematic review: kidney transplantation compared with dialysis in clinically relevant outcomes. Am J Transplant. 2011;11(10):2093-2109.

Wiebe C, Pochinco D, Blydt-Hansen TD, et al. Class II HLA epitope matching—a strategy to minimize de novo donor-specific antibody development and improve outcomes. *Am J Transplant*. 2013;13:3114–3122.

Wiebe C, Kosmoliaptsis V, Pochinco D, et al. HLA-DR/DQ molecular mismatch: A prognostic biomarker for primary alloimmunity. *Am J Transplant*. 2019;19:1708–1719.

Notes
