# Supplementary material for: Adding epitope compatibility to deceased donor kidney allocation criteria: recommendations from a pan-Canadian online public deliberation
Source: BMC Nephrol. 2023 Jun 9;24:165. doi: 10.1186/s12882-023-03224-z (PMC10255937; doi:10.1186/s12882-023-03224-z)
Supplement: Supplementary file 2 — Additional file 2. [file 12882_2023_3224_MOESM2_ESM.docx]

**Additional file 2:**

**Expert speaker perspectives, summary of main presentation points, and brief biography**

NB: Expert speaker names, headshots, and biographies were shared in advance of Session 1 with participants, but the summary presentation points were not provided. These are included here to give an overview of the range of topics covered in these presentations.

**Dr. James Lan – clinician perspective**, which will cover:

1. *Current state of kidney allocation decision-making in BC (i.e., wait list system)*
2. *Clinical perspective of poorly matched transplant outcomes (e.g., rejection, more sensitization for young recipients that are likely to need multiple transplants)*
3. *Uncertainty around wait times with epitope matching*

Dr. Lan is an Assistant Professor jointly appointed to the UBC Department of Pathology and Laboratory Medicine and the Department of Medicine, Division of Nephrology. He is the Medical Director of the HLA Laboratory in Vancouver, and a transplant nephrologist at the Vancouver General Hospital.

**Dr. Christy Simpson – bioethicist perspective**, which will cover:

1. *Framing for organ allocation from an ethics, values-based perspective*
2. *Need to be alert to possible inequities that may arise in relation to any adjustments or changes to the allocation process*
3. *Need to consider and reflect on a range of outcomes, of which improved kidney function is one outcome but not the only one we need to keep in mind*

Dr. Simpson is an Associate Professor and (now former) Head of the Department of Bioethics, Faculty of Medicine, Dalhousie University. She is also the Department’s Coordinator of the Ethics Collaborations Team which provides ethics support for Nova Scotia Health, IWK Health, and the Nova Scotia Health Ethics Network.

**Dr. Mary Wilson – medicine woman, Indigenous knowledge keeper and elder perspective**, which will cover:

1. *Everything is inter-related*
2. *A kidney is a gift of life*
3. *How traditional medicine is passed on*

Dr. Wilson is a spiritual healer, wisdom & Knowledge Keeper living in Winnipeg, MB. Mary focuses on rebuilding community and individual strength and independence through a wide range of therapies, practices and guided spiritual programs to ultimately create a better life and place for everyone.

**Teresa Atkinson (supported by Jen Desjardins) – patient perspective**, which will cover:

1. *Leaning in to hope to survive CKD*
2. *Dialysis experiences*
3. *Transplant experiences*

Teresa has lived with kidney disease for 35 years and has spent more time on dialysis than living with a successful transplant. After a 17-year wait on dialysis, Teresa was lucky enough to receive her 3^rd^ miracle transplant from the highly sensitized list.

Jen was diagnosed with kidney failure when she was 16, and eventually received a kidney from her mother. Nine years later, she found out she had cancer. She had to stop taking the immunosuppressants to be able to fight the cancer, but this led her body to reject her mother's transplanted kidney. Radical surgery was done to remove the cancer, and she had to be cancer-free for 5 years before she could go back on the transplant wait list. After the long wait, she ended up receiving a second kidney from her cousin. This year is her 15^th^ transplant anniversary and 21 years cancer-free.
