# Supplementary material for: Adding epitope compatibility to deceased donor kidney allocation criteria: recommendations from a pan-Canadian online public deliberation
Source: BMC Nephrol. 2023 Jun 9;24:165. doi: 10.1186/s12882-023-03224-z (PMC10255937; doi:10.1186/s12882-023-03224-z)
Supplement: Supplementary file 3 — Additional file 3. [file 12882_2023_3224_MOESM3_ESM.docx]

**Additional file 3:**

**Deliberative questions and accompanying narrative scenario**

Question 1. How can epitope-based allocation be implemented in a way that is fair for

transplant candidates?

*Current wait list system:*

*George has just joined the wait list to receive a kidney transplant in the current*

*system. Because he has a commonly occurring blood type, he is in the easy-to-match*

*category. He is told that there is a good chance that he will be able to find a kidney that*

*he is compatible with without a long wait. He will probably have to wait about 2 years*

*for those who are already on the wait list to be matched to a kidney first. It could be 6*

*months shorter or 6 months longer than this (2 years, +/- 6 months), but the transplant*

*team will be in touch when they have any news. In the meantime, George will have to*

*remain on dialysis. For him, this means going to the local clinic 3 times a week to*

*receive dialysis treatment for 4 hours each time.*

*Sarah has also joined the wait list. She is more difficult to match because she has*

*blood type B, and has been told that it will be more challenging to find a kidney for her.*

*She is told that it could be a fairly long wait for a kidney to become available that she is*

*compatible with, but there is hope that they will find a good match for her. She is added*

*to an existing wait list with others in this more difficult-to-match group, and she learns*

*that she has about 5 years to wait for a kidney. It could be 1-2 years shorter or longer*

*than this (5 years, +/- 1 or 2 years) because Sarah is in this more difficult-to-match*

*group, but the transplant team will be in touch when they have any news. In the*

*meantime, Sarah will have to remain on dialysis. Sarah must also visit the dialysis clinic 3*

*times a week for 4 hours at a time.*

*Although George and Sarah know roughly how long they have to wait for a kidney, there*

*is uncertainty about how well or for how long the kidney they are offered will function.*

*This is because the current wait list system assigns the next available kidney to the next*

*compatible candidate on the wait list.*

*Epitope compatibility system:*

*A new epitope compatibility system for allocating kidneys has been adopted for*

*candidates in several transplant regions in Canada. George and Sarah live in one of the transplant regions that have opted into this new allocation system.*

*Although George and Sarah remain just as easy- or more difficult-to-match on the same factors as before, they will now also need to wait for the most-epitope compatible kidney.* *They find out that all candidates with the same clinical factors as themselves are in a pool, instead of on an ordered wait list, and they will all be considered each time a kidney with the same clinical factors becomes available. Since the transplant team will try to match the next available kidney to the person who is most likely to have the best outcome, it is hard to give an accurate prediction about how long the wait will be. George and Sarah must still remain on dialysis while the wait for their transplant, which means 3 visits to the dialysis clinic a week for 4 hours each time.*

*George is told that there is quite a bit of uncertainty about how long he will have to wait for a kidney. Because the entire pool of candidates is assessed for epitope compatibility each time a kidney becomes available, the best guess they can give him is that the wait could be 1-4 years. But, this will be for a better-matched kidney. For Sarah, there is even more uncertainty because of her blood type. She is told that she might wait less than 5 years, but it could be up to 10 years for a better-matched kidney.*

*Although there is greater uncertainty about how long they will have to wait for a kidney, they are more likely to be offered a kidney that functions better and for longer. This is because the epitope compatibility system assigns the next available kidney to the best matched candidate.*

Question 2. What are important considerations in the way kidney allocation policies and decisions are made?
